# Level of mortality risk for babies born preterm or with a small weight for gestation in a tertiary hospital of Nepal

**DOI:** 10.1186/s12889-015-2232-1

**Published:** 2015-09-10

**Authors:** Ashish KC, Johan Wrammert, Viktoria Nelin, Uwe Ewald, Robert Clark, Mats Målqvist

**Affiliations:** International Maternal and Child Health, Department of Women’s and Children’s Health, Uppsala University, Uppsala, Sweden; United Nation’s Children’s Fund, Nepal Country Office, UN House Pulchowk, Lalitpur, Nepal; Latter Day Saints Charities, Salt Lake City, Utah USA

## Abstract

**Background:**

Globally, 15 million babies were born prematurely in 2012, with 37.6 % of them in South Asia. About 32.4 million infants were born small for gestational age (SGA) in 2010, with more than half of these births occurring in South Asia. In Nepal, 14 % of babies were born preterm and 39.3 % were born SGA in 2010. We conducted a study in a tertiary hospital of Nepal to assess the level of risk for neonatal mortality among babies who were born prematurely and/or SGA.

**Methods:**

This case–control study was completed over a 15-month period between July 2012 and September 2013. All neonatal deaths that occurred during the study period were included as cases and 20 % of women with live births were randomly selected as referents. Information on potential risk factors was taken from medical records and interviews with the women. Logistic regression analyses were conducted to determine the level of risk for neonatal mortality among babies born preterm and/or SGA.

**Results:**

During this period, the hospital had an incidence of preterm birth and SGA of 8.1 and 37.5 %, respectively. In the multivariate model, there was a 12-fold increased risk of neonatal death among preterm infants compared to term. Babies who were SGA had a 40 % higher risk of neonatal death compared to those who were not. Additionally, babies who were both preterm and SGA were 16 times more likely to die during the neonatal period.

**Conclusions:**

Our study showed that the risk of neonatal mortality was highest when the baby was born both preterm and SGA, followed by babies who were born preterm, and then by babies who were SGA in a tertiary hospital in Nepal. In tertiary care settings, the risk of mortality for babies who are born preterm and/or SGA can be reduced with low-cost interventions such as Kangaroo Mother Care or improved management of complications through special newborn care or neonatal intensive care units. The risk of death for babies who are born prematurely and/or SGA can thus be used as an indicator to monitor the quality of care for these babies in health facility settings.

**Clinical trial registration:**

ISRCTN97846009

## Background

In 2012, 2.9 million neonatal deaths (deaths in the first 28 days after birth) occurred globally; of these, almost 34 % (1 million) were directly caused by complications due to preterm birth (before 37 gestational weeks) [1]. Additionally, preterm birth was a risk factor for approximately 1.47 million neonatal deaths, which were directly due to other causes (e.g., infection) [2].

In 2012, an estimated 15 million babies (11.3 % of live births) worldwide were born preterm, about 13 million of these infants survived beyond the first month of life [2–5]. A large proportion of these preterm births (37.6 %) occurred in South Asia, for a prevalence rate of 13.3 % among all live births in the region [6].

A meta-analysis on the association between neonatal mortality and preterm birth in East Africa showed that babies born at <34 weeks gestation had a 58-fold increased risk for neonatal death than babies who were born at term. Further, babies who were born between 34 and 36 weeks of gestation had 3.2 times higher risk for neonatal death than babies who were born at term [7].

An infant is considered small for gestational age (SGA) when they are born with a birth weight below the tenth percentile according to a particular gestational age and sex-specific reference [8]. SGA can occur among infants who grew healthily in utero, but were naturally small. Alternatively, SGA can occur among infants who suffered from intrauterine growth restriction, which can be caused by a number of factors including placental insufficiency, environmental exposures, nutritional factors, etc. [9].

In 2010, 32.4 million SGA infants were born worldwide, of which 2.8 million were also preterm [3]. Approximately 706,200 deaths were attributable to SGA, globally. More than half of SGA births occurred in South Asia, where the prevalence of SGA was 44.5 % and the prevalence of preterm and SGA births was 2.9 % in 2010 [6]. In 2012, more than 80 % of neonatal deaths in Sub-Saharan Africa and South Asia were among small babies (65 % attributable to preterm birth and 19 % to term-SGA)[6]. Two-thirds of neonatal deaths among SGA infants were of term, low birth weight babies [2].

Nepal, a low-income country in South Asia, has a neonatal mortality rate of 24 per thousand live births, with more than half of these deaths caused by preterm birth-related complications [1]. In 2010, 14 % of babies were born preterm and 39.3 % were SGA at birth in Nepal [6]. By determining the level of risk for neonatal death in babies born prematurely and/or SGA in a hospital setting in Nepal, we can provide evidence of the need for improved quality of care for these infants through investments in evidence-based, low-cost interventions to increase survival. Therefore, we conducted this assessment to identify the association between neonatal mortality and preterm birth and/or SGA among babies born in a tertiary hospital of Nepal.

### Methods

#### Design

We conducted a case–control study, nested within a larger hospital-based prospective cohort study aiming to evaluate the impact of a simplified neonatal resuscitation protocol on perinatal outcomes. For the purpose of the larger study, a reference population was selected from the source population (all admissions to the hospital for delivery) to assess the change in perinatal outcomes over a specified period of time. The reference population of the larger study was a randomly selected 20 % of the women delivering in the hospital. This random selection was done using a lottery technique at the time of admission. For the purpose of this study, all live births from the reference population were selected to be in the referent population and all neonatal deaths occurring during the study period were included in the case population. Any neonatal death that occurred within the referent population was removed from that population and re-classified into the case population. The sample size for the study was based on the larger study and calculated to detect a 20 % reduction in perinatal mortality, with a statistical power of 80 % and level of significance at 5 % [10]. This case–control study was conducted from July 1, 2012 to September 30, 2013.

#### Setting

We conducted this study in a tertiary hospital, Paropakar Maternity and Women’s Hospital, located in Kathmandu, Nepal. This government-funded hospital has 415 beds, with 407 staff equipped to provide comprehensive obstetric and gynecological services. The hospital has three delivery units, as well as a Kangaroo Mother Care unit, special newborn care unit and neonatal intensive care unit for the care of small and sick babies born there. Around 22,000 deliveries take place annually, with an incidence of preterm birth and low birth weight at 9 and 11 %, respectively in 2012, and a neonatal mortality rate of 9 per thousand live births [11].

As part of the larger study evaluating the impact of neonatal resuscitation protocol implementation, the study received approval from the Hospital’s Institutional Review Committee, the Nepal Health Research Council (Reg. No. 37/2012) and the Ethical Review Board of Uppsala University (dnr 2012/267). The study was registered as clinical trial, ISRCTN 97846009 [10]. Written consent was taken from all women who participated in the study.

#### Participants

All women with a neonatal death occurring during their stay in the hospital, throughout the study period were included as cases. Randomly selected women in the reference population (from original study), with live births were included in the referent population and were followed upto 28 days of birth. Any antepartum or intrapartum stillbirths occurring in the referent population were excluded from the study. Any neonatal death occurring in the referent population was excluded from this group, re-categorized, and included in the case population.

#### Data collection

A surveillance system was set up to collect socio-demographic, obstetric and postpartum information from the women in the case and referent populations at the admission, delivery and postnatal units. A surveillance team member at the admission unit collected information from the women who were admitted to the hospital for delivery. The team randomly selected 20 % of women admitted to the hospital using a lottery technique. The surveillance team members at the delivery and postnatal units followed the referent women until discharge and followed up on the birth outcome through telephone interview conducted 28 days after delivery. The surveillance team at the delivery and postnatal units also collected information on the case population, i.e. all neonatal deaths that occurred in the hospital. Information about the case and referent populations was taken from the women’s individual client journals, including demographic characteristics, obstetric history, intrapartum clinical progress and outcomes, and neonatal information. For certain socio-economic information, short interviews were completed with the women from the case and referent populations.

#### Variables

***Neonatal mortality****:* Death of an infant from the time of birth until 28 days.

***Preterm birth****:* Babies born before 37 completed weeks of gestation, estimated by the date of the mother’s last menstrual period or based on clinical examination of the newborn.

***Term birth****:* Babies who were born at, or after, 37 completed weeks of gestation, estimated by the mother’s last menstrual period or based on clinical examination of the newborn.

***Small for gestational age****(****SGA****):* Babies whose birth weight was less than the 10th percentile according to the appropriate gestational age and sex-specific reference population standards [8].

***Appropriate for gestational age****(****AGA****):* Babies whose birth weight was greater than or equal to the 10th percentile according to the appropriate gestational age and sex-specific reference population standards [8].

***Low Birth Weight****(****LBW****):* Babies who weighed less than 2500 grams at the time of birth.

***Wealth quintile****:* The wealth index is a measure of socioeconomic position, used in nationally representative health surveys (Demographic Health Surveys) to compare the socio-economic inequalities [12, 13]. During the interviews with mothers, data was collected on ownership of durable assets (e.g. car, refigerator, bicycle, radio, television), housing characteristics (e.g. number of rooms, dwelling floor and roof materials, toilet facilities) and access to services (e.g. electricity supply, drinking water source). Using the scores from the first principal component analysis, a wealth index (asset index) was contructed. Based on the value of the index, individuals were sorted and established to create cut-off values for percentiles within the population. These quintiles were then ranked from bottom to top as poorest, poorer, middle, richer and richest [14].

***Ethnicity****:* The group within the social hierarchical system of Nepal to which the women’s family belongs [15].

***Parity***: Number of times a woman has given birth after the age of viability, i.e. 22 weeks, including both live and still births [16].

***Antenatal care attendance****:* The number of antenatal care visits that a woman went to in order to receive antenatal care from a skilled health worker.

***Obstetric complication during pregnancy***: Any complication that a woman had during the pregnancy period [17], including the following:

*Antepartum hemorrhage:* Excessive vaginal bleeding occurring before the onset of labor.

*Hypertensive disorder during pregnancy:* Classified by maternal diastolic blood pressure greater than or equal to 90 mmHg in two different recordings, at least 4 h apart.

*Multiple pregnancies*: When a woman was pregnant with more than one fetus.

*Medical disorder:* When a woman had any of the following: diabetes mellitus, severe anemia (Hb <7 gm/L), epilepsy, or other serious medical condition during pregnancy.

***Obstetric complication during delivery****:* Any complication that a woman had during the intrapartum period [17], including the following:

*Hypertensive disorder:* Classified by maternal diastolic blood pressure greater than or equal to 90 mmHg in two separate recordings.

*Mal-presentation*: Presentation of the fetus in any position besides vertex, i.e. with the top of the head appearing first.

*Prolonged labor*: When cervical dilation did not move beyond 4 cm after 8 h of regular contractions, or if cervical dilation was to the right of the alert line on the partogram.

*Prolapsed cord*: Characterized by the presence of the umbilical cord in the birth canal below the fetal presenting part, or at the vagina following the rupture of membranes.

#### Data analysis

The demographic, social and obstetric characteristics of the case and referent populations were compared using a Pearson’s chi-square test, Wilcoxon rank-sum *t*-test or Fisher’s exact test to assess whether there was a difference (*p* < 0.05) between the two groups.

For comparison of the demographic, social and obstetric characteristics of the case and referent populations, categorical variables were created. Maternal age was categorized into 5-year intervals including ≤20, 21–25, 26–30, and >30 years; maternal education was categorized as less than 6 years of education or greater than or equal to 6 years of education; ethnicity was classified into six groups as Brahmin/Chhetri (hill and terai), relatively advantaged Janajatis (Newar, Gurung, Thakali), disadvantaged Janajatis, Dalit (hill and terai), non-Dalit terai, and Muslims; wealth was classified into five population quintiles: poorest, poorer, middle, richer and richest; parity was classified into three groups: primiparous, multiparous (1–2) and multiparous (3 or more); antenatal care attendance was classified as having attended any antenatal care or none; obstetric complications during pregnancy were classified as having any or having none; obstetric complications during the intrapartum period were also classified as having any versus none; the number of babies was categorized as multiple pregnancy or not (i.e., singleton); the sex of the baby as male or female; mode of delivery as vaginal, instrumental or cesarean section; gestational age at birth was classified as term versus preterm; and size according to gestational age was classified as SGA or AGA. We also created a binary variable grouping babies who were born both preterm and SGA versus those who were neither.

Univariate logistic regression analysis was done to test the association between neonatal death and demographic, social and obstetric characteristics of the women and babies that showed differences (*p* < 0.01) between the case and referent populations. Three different multivariable models were created to assess the level of association of neonatal mortality with preterm and/or SGA after adjusting for maternal age, maternal educational status, antenatal care attendance, wealth status, complication during the intrapartum period, mode of delivery, parity and multiple pregnancy. The first multivariable model assessed the level of association between neonatal mortality and preterm birth compared to term; the second multivariable model assessed the level of association between neonatal mortality and being born SGA compared to babies born AGA; and, the third model assessed the level of association between neonatal mortality and being born both preterm and SGA compared to being born only preterm, or only SGA, or neither.

We used the multiple imputation method to deal with data missing at random from the case or referent populations for the demographic, social, and/or obstetric variables [18].

## Results

**Fig. 1:**
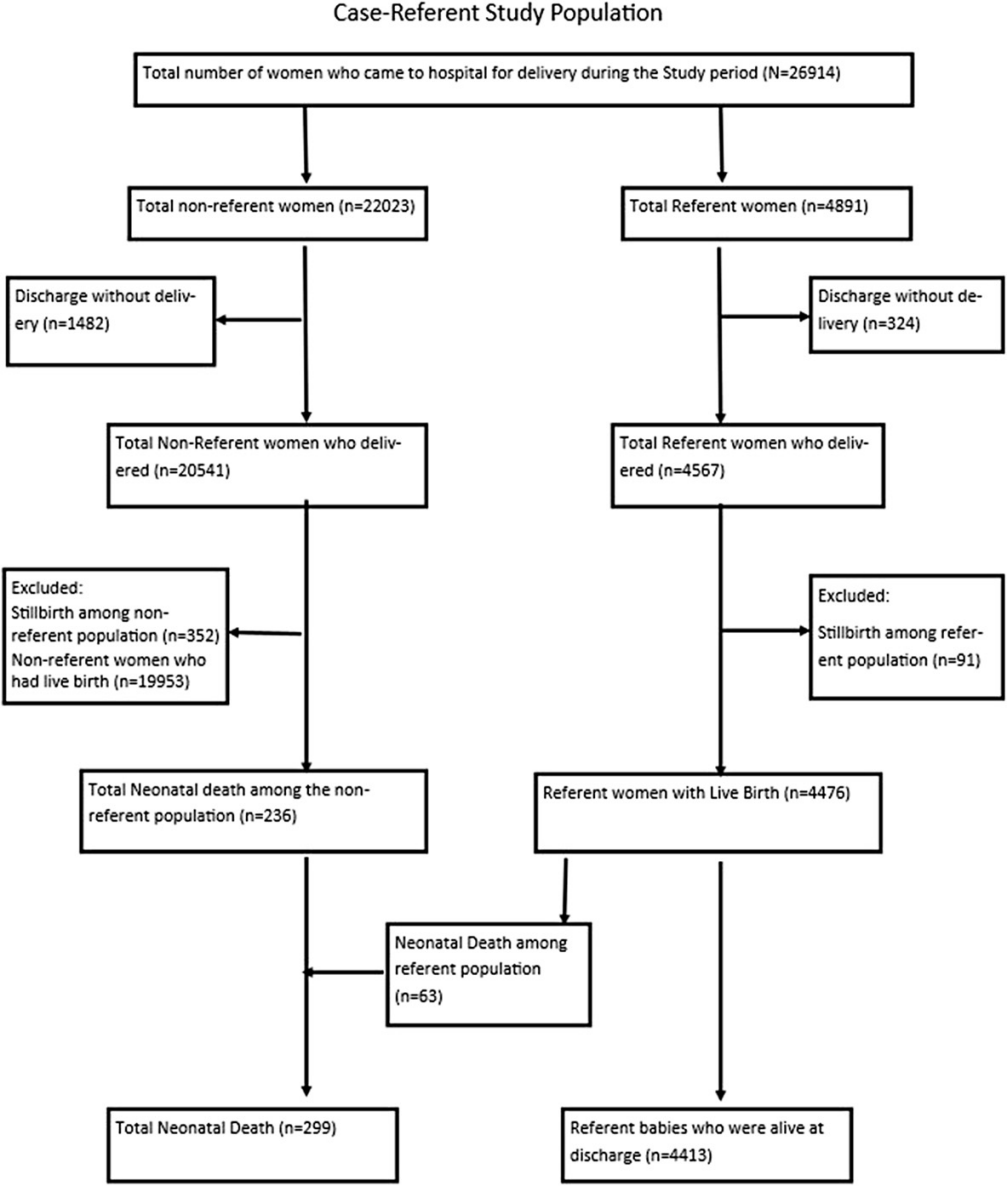
Flow chart of study participants

During the study period, there were 25,108 women who delivered in the hospital and a total of 299 neonatal deaths, giving a neonatal mortality rate of 11.9 per thousand live births. A total of 4,413 referent babies were alive at the time of their discharge from the hospital and thus included in the referent population (Fig. 1). During this time, the hospital had an incidence rate of preterm birth and SGA of 8.1 and 37.5 %, respectively.

The mean maternal ages of the case and referent populations were 24.3 and 23.7 years, respectively, which was significantly different between the two groups (*p* < 0.001). Women with less education, who were from the poorest family, multiparous, or who had no antenatal care had more neonatal deaths (*p* < 0.001). Similarly, there were more neonatal deaths among women with obstetric complications during the intrapartum period than those women without (*p* < 0.001). There were also more neonatal deaths among babies who were born prematurely, with a low birth weight or SGA than babies who were born at term, with a normal birth weight or AGA (*p* < 0.001) (Table 1).

**Table 1:** Demographic, social and obstetric characteristics of live births (referent population) and neonatal deaths (case population)

Characteristics	Live births (n = 4413)	Neonatal deaths (n = 299)	*p*-value*
Maternal age (years)			
Mean ± standard deviation	23.7 ± 4.4	24.3 ± 5.6	*p* = 0.71
Median (interquartile range)	23 (20–26)	23 (20–28)	*p* = 0.63
Intervals:	n (%)	n (%)	
≤ 20	1204 (27.3)	85 (28.4)	
21–25	1931 (43.8)	120 (40.1)	
26–30	961 (21.8)	49 (16.4)	
> 30	317 (7.2)	45 (15.1)	*p* < 0.001
Ethnicity			
Brahmin/Chhetri (Hill and Terai)	1710 (38.7)	110 (36.8)	
Relatively advantaged Janajatis	804 (18.2)	52 (17.4)	
Disadvantaged Janajatis	1274 (28.9)	84 (28.1)	
Non-Dalit Terai	363 (8.2)	31 (10.4)	
Dalit (Hill and Terai)	230 (5.2)	16 (5.4)	
Muslim	32 (0.7)	6 (2.0)	*p* = 0.174
Maternal education			
Six years of education or less	70 (23.4)	1448 (32.8)	
More than 6 years of education	229 (76.6)	2965 (67.2)	*p* = 0.001
Wealth index (quintiles)^a^			
Poorest	768 (18.7)	67 (39.2)	
Poorer	802 (19.6)	26 (15.2)	
Middle	857 (20.9)	29 (17.0)	
Richer	830 (20.2)	29 (17.0)	
Richest	843 (20.6)	20 (11.7)	*p* < 0.001
Parity			
Primiparous	2385 (54.0)	146 (48.8)	
Multiparous (1–2)	1845 (41.8)	118 (39.5)	
Multiparous (≥3)	183 (4.1)	35 (11.7)	*p* < 0.001
Antenatal care attendance			
Yes	3857 (87.4)	174 (58.2)	
No	556 (12.6)	125 (41.8)	*p* < 0.001
Obstetric complication(s) in antepartum period			
No	3267 (74.0)	233 (77.9)	
Yes	1146 (26.0)	66 (22.1)	*p* < 0.151
Multiple pregnancy			
No	4378 (99.2)	283 (94.6)	
Yes	35 (0.8)	16 (5.4)	*p* < 0.001
Obstetric complication(s) in intrapartum period			
No	3923 (88.9)	194 (64.9)	
Yes	490 (11.1)	105 (35.1)	*p* < 0.001
Mode of delivery			
Vaginal	3418 (77.5)	195 (65.2)	
Cesarean section	995 (22.5)	104 (34.8)	*p* < 0.001
Sex			
Male	2333 (52.9)	176 (58.9)	
Female	2080 (47.1)	123 (41.1)	*p* = 0.074
Preterm (<37 weeks)			
No	4079 (92.4)	116 (38.8)	
Yes	334 (7.6)	183 (61.2)	*p* < 0.001
Birth weight (grams)			
Mean ± standard deviation	2934.2 ± 473.4	1853.7 ± 796.8	*p* < 0.001
Median (interquartile range)	3000 (2600–3250)	1650 (1200–2400)	*p* < 0.001
	n (%)	n (%)	
Small for gestational age (<10th percentile)			
No	2772 (62.8)	156 (52.2)	
Yes	1641 (37.2)	143 (47.8)	*p* < 0.001
Preterm and Small for gestational age			
No	4349 (98.5)	239 (79.9)	
Yes	64 (1.5)	60 (20.1)	*p* < 0.001

*p-value was determined by Pearson’s chi-square test, Wilcoxon rank-sum t-test or Fisher’s exact test

^a^There are some missing values for the wealth index variable: 313 missing among the live birth group (referent population) and 128 missing in the neonatal death group (case population)

Univariate logistic regression analysis assessing the likelihood for neonatal mortality based on maternal demographic characteristics showed that older woman, those with less education (6 years or less) or from the poorest families had an increased risk for neonatal mortality. For analyses based on obstetric characteristics, those women who were multiparous, who had no antenatal care from a skilled provider, had obstetric complications during the intrapartum period, who had a multiple pregnancy or Cesarean section also had a higher risk of neonatal mortality. The level of risk for neonatal mortality increased by 50 % if the baby was SGA, for preterm babies the risk increased 17-fold, and there was a 19-fold increased risk if the baby was both preterm and SGA (Table 2).

**Table 2:** Logistic regression analyses for likelihood of neonatal death based on selected maternal and infant characteristics

	Crude Odds Ratio (cOR)^a^	95 % CI
Maternal age (years)		
Maternal age	Ref	
Increase in age	1.02	1.0–1.1
Maternal education		
More than 6 years of education	Ref	
Education of less than 6 years	1.6	1.2–2.1
Wealth status^b^		
Non-poor	Ref	
Poor	1.4	1.0–1.8
Parity		
Primi-parous	Ref	
Multi-parous	1.4	1.2–1.5
Antenatal care attendance		
Yes	Ref	
No	5.0	3.9–6.4
Multiple pregnancy		
No	Ref	
Yes	7.1	3.9–12.9
Obstetric complication during intrapartum period		
No	Ref	
Yes	4.3	3.4–5.6
Mode of delivery		
Vaginal delivery	Ref	
Cesarean section	1.8	1.4–2.4
Preterm (<37 weeks of gestation)		
No	Ref	
Yes	17.1	11.7–24.8
Small for gestational age (<10th percentile)		
No	Ref	
Yes	1.5	1.2–2.0
Preterm and Small for Gestational Age		
No	Ref	
Yes	19.3	14.9–25.0

^a^Univariate logistic regression analysis done to test the association between neonatal death and characteristics of the women and babies that showed differences (*p* < 0.01) among the case and referent populations

^b^The women from the poorest wealth quintile were categorized as poor and the remaining women in the poorer to richest quintiles were categorized as non-poor

In the multivariable logistic regression models, the following characteristics were adjusted for, as they were different between the case and referent populations: maternal age, maternal educational status, wealth status, parity, antenatal care attendance, multiple pregnancy, obstetric complication during the intrapartum period, and mode of delivery. After adjusting for these potential confounders, the likelihood for neonatal death was 12 times higher for babies who were preterm than those who were born at term, 40 % higher for SGA compared to AGA infants and 16 times higher for babies who were both preterm and SGA (Table 3).

**Table 3:** Multivariable regression analysis for risk of neonatal death for babies born preterm, small weight for gestational age (SGA) or both

	Adjusted Odds Ratio^a^	95 % CI
Preterm (<37 weeks of gestation)		
No	Ref	
Yes	12.4	8.1–18.9
SGA (<10th percentile)		
No	Ref	
Yes	1.4	1.1–1.8
Preterm and SGA		
No	Ref	
Yes	16.2	12.3–21.3

^a^Multivariable logistic regression analysis for the likelihood of neonatal death, adjusted for maternal age, maternal educational status, antenatal care attendance, wealth status, complication(s) during the intrapartum period, mode of delivery, parity, and multiple pregnancy

## Discussion

Our study showed that the risk of neonatal mortality was highest among babies born both preterm and SGA, followed by babies born preterm, and then by SGA babies in Nepal. Babies who are born prematurely or SGA have an increased risk for hypothermia, infection, respiratory distress syndrome (RDS), intracranial hemorrhage, necrotizing enterocolitis, retinopathy of prematurity, neurodevelopmental impairment and mortality [9, 19, 20]. These complications could potentially be prevented, or minimized, with interventions like Kangaroo Mother Care and extra-support for feeding, case management of babies with signs of infection, safe oxygen management and supportive care for RDS, hospital care of babies with RDS, use of continuous positive airway pressure (CPAP) and surfactant, or intensive neonatal care [21–24].

There were some potential limitations within this study. First, there may have been some bias within this study, such as the failure of the health worker to correctly assess the medical condition during clinical examination at admission or during delivery. Second, the gestational age estimate we used was based on the last maternal menstrual period, so there may have been some recall bias from the women. Third, the weight for gestational age references used were based on a US national population, which are likely different from what the Nepal national population references would be. Fourth, the telephone follow ups were only done for referent population, so the neonatal death occurring in the non-referent population (80 %) after discharge were missed, which could potentially result in under-reporting of neonatal mortality due to prematurity or small for gestational age. Finally, this is a hospital-based study, and the population coming to this tertiary referral hospital may not be representative of the larger population in Nepal. However, the objective of this paper was to assess the level of risk for neonatal death among infants who were either preterm and/or SGA in a tertiary hospital setting, so that in the future, the quality of care provided for these infants can be improved.

A pooled analysis conducted using data from low- and middle-income countries to determine the risk for neonatal mortality among preterm and SGA infants showed that babies who were preterm had a 6.8 times higher risk for death than babies born term; SGA babies had a 1.83 times increased risk for neonatal death compared to AGA; and babies who were both had a 15-fold increased risk for neonatal mortality [25]. Our study showed the level of risk for neonatal mortality among these same groups to be higher than the pooled analysis conducted in other low- and middle-income countries.

In Nepal, more than half of total deliveries take place at health institutions, and care seeking for sick and small babies at referral hospitals has increased in the last 10 years [26, 27]. Together with this increased use of referral hospitals for delivery and postnatal care, the findings of increased likelihood for neonatal death among high-risk infants from our study, done in a referral hospital setting, indicate that interventions for reducing preterm- and/or SGA-complication related mortality have not yet been extensively implemented. As a country, Nepal has committed to reduce the neonatal mortality rate to 10 (or less) per thousand live births by 2035 at the World Health Assembly 2014 as part of the Every Newborn Action Plan [28]. Based on our estimates, the current proportion of neonatal deaths due to preterm- and/or SGA-related complications has to be reduced by half, in order to meet this goal.

## Conclusion

To our knowledge, this is the first study done in Nepal with the aim to determine the level of risk for neonatal death among babies who were born prematurely and/or SGA in a tertiary health facility. The increased risk for neonatal death among preterm and/or SGA infants together with the increased trend for institutional delivery, reveals the need for furthered investment in the scale up of evidence-based interventions such as Kangaroo Mother Care or antenatal corticosteroids, as well as the improvement of special newborn care and neonatal intensive care units. Periodic analysis on the level of risk for neonatal mortality among preterm and SGA babies in health facilities can provide a reflection of the efficiency of the implementation of such interventions to reduce preterm- and SGA-related deaths.
